# Bioactivity of periodontal ligament stem cells on sodium titanate coated with graphene oxide

**DOI:** 10.1038/srep19343

**Published:** 2016-01-14

**Authors:** Qi Zhou, Pishan Yang, Xianlei Li, Hong Liu, Shaohua Ge

**Affiliations:** 1 Shandong Provincial Key Laboratory of Oral Tissue Regeneration, School of Stomatology, Shandong University, Jinan, China; 2 Department of Periodontology, School of Stomatology, Shandong University, Jinan, China; 3 State Key Laboratory of Crystal Materials, Shandong University, Jinan, China; 4 Center of Bio and Micro/Nano Functional Materials, State Key Laboratory of Crystal Materials, Shandong University, Jinan, China

## Abstract

As a biocompatible and low cytotoxic nanomaterial, graphene oxide (GO) has captured tremendous interests in tissue engineering. However, little is known about the behavior of dental stem cells on GO. This study was to evaluate the bioactivity of human periodontal ligament stem cells (PDLSCs) on GO coated titanium (GO-Ti) substrate *in vitro* as compared to sodium titanate (Na-Ti) substrate. By scanning electron microscope (SEM), confocal laser scanning microscope (CLSM), methylthiazol tetrazolium (MTT) assay, alkaline phosphatase (ALP) activity, quantitative real-time polymerase chain reaction (qRT-PCR) and western blot analysis, we investigated the attachment, morphology, proliferation and osteogenic differentiation of PDLSCs on these two substrates. When seeded on GO-Ti substrate, PDLSCs exhibited significantly higher proliferation rate, ALP activity and up-regulated gene expression level of osteogenesis-related markers of collagen type I (COL-I), ALP, bone sialoprotein (BSP), runt related transcription factor 2 (Runx2) and osteocalcin (OCN) compared with those on Na-Ti substrate. Moreover, GO promoted the protein expression of BSP, Runx2 and OCN. These findings suggest that the combination of GO and PDLSCs provides a promising construct for regenerative dentistry.

Graphene, the parent of all graphitic forms, is an atomic-thick sheet of carbon atoms arranged in two-dimensional (2D) honeycomb structure with high electric and thermal conductivities, high mechanical strength, and excellent optical properties^1^. Due to its unique physicochemical properties, graphene has been considered as a promising candidate in many applications and technological aspects such as nanoelectronics, polymer composites, sensors, batteries, fuel cells and super capacitors^1^,^2^,^3^,^4^,^5^,^6^. In the area of biomedicine, graphene and its derivatives such as graphene oxide (GO) have been highly anticipated to provide unique and new opportunities for the developments of novel biosensors^7^,^8^,^9^,^10^, nanocarriers for drug and gene delivery^11^,^12^,^13^, cell imaging and photo-therapy of cancer^14^,^15^,^16^. GO has been used as coating material of substrates to culture stem cells, such as being covered onto the substrates of glass slides, polydimethylsiloxane (PDMS), chitosan, and gelatin-hydroxyapatite (GHA)^17^,^18^,^19^,^20^,^21^. The results proved that GO played an important role in promoting the differentiation of stem cells into osteoblasts. Besides, the studies of cultivating NIH-3T3 fibroblasts, MC3T3-E1 cells, and A549 on GO scaffolds have further demonstrated that GO influences cell proliferation and differentiation positively^22^,^23^,^24^.

Implant therapy is a reliable treatment for replacement of missing teeth, because of the high predictability of success. Surface properties of titanium implants including surface composition, hydrophilicity and roughness play important roles in implant- osseointegration and implant-tissue interaction^25^. The surface chemical composition and roughness affect the protein absorption, cell attachment and hydrophilicity of the surface^25^. Therefore, finding a way to modify the surface of dental implants to promote osseointegration might help the clinicians to maximize the success rate of implants and diminish the complications that can be encountered after their placement. La *et al*. reported that GO-coated titanium substrate carrying with bone morphogenetic protein-2 (BMP-2) could up-regulate osteogenic differentiation of bone marrow derived mesenchymal stem cells (BMMSCs) compared with BMP-2 titanium *in vitro*^26^. However, its effect on bioactivities of dental stem cells remains unknown. Human periodontal ligament stem cells (PDLSCs) are promising dental stem cells and are used as an alternative to human BMMSCs for determining the osteogenic potential of titanium films and for assessing osseointegration in titanium implants^27^. PDLSCs have potentials for bone formation on various titanium surfaces, which is demonstrated by the expression of bone forming related proteins^28^. Moreover, surface roughness and hydrophilicity of titanium can affect differential Wnt pathways and signaling molecules, targeting the osteogenic differentiation of PDLSCs^29^. A recent study demonstrated that a composite film of GO could enhance cell adhesion, proliferation and mesenchymal phenotype expression of PDLSCs^30^. In spite of these studies, the behavior of dental stem cells, especially the response of PDLSCs to GO-coated titanium (GO-Ti) substrate is not fully understood.

In this study, we fabricated GO-Ti substrate as a cell culture platform. Then, we analyzed the effects of GO-Ti substrate on cell morphology, proliferation and osteogenic differentiation potentials of PDLSCs.

## Results

### Fabrication and characterization of GO films

**Figure 1:**
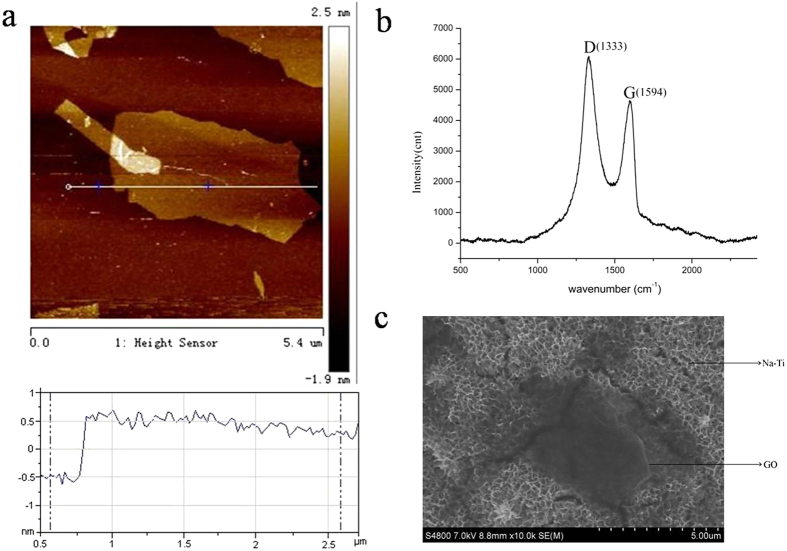
Fabrication and characterization of GO films. (**a**) AFM image and quantitative analysis of the depth profile of GO-Ti film. (**b**) Raman spectra analysis of GO-Ti film. (**c**) SEM image of GO-Ti film.

The GO sheets were characterized by atomic force microscopy (AFM). The AFM profiles (Fig. 1a) showed that GO sheets on the quartz substrate were about 1 nm in height, which suggests that the GO sheets are monolayer. The Raman spectrum of the GO-Ti sheets (Fig. 1b) displayed two prominent peaks at 1333 and 1594 cm^−1^, which correspond to the well-documented D and G band of graphene oxide, respectively^31^. Scanning electron microscope (SEM) image of GO-Ti (Fig. 1c) showed the GO sheets of different sizes were deposited on the titanium substrate. The wrinkles could be clearly seen due to its small thickness, which was consistent with the AFM data.

### Characteristics of human PDLSCs

**Figure 2:**
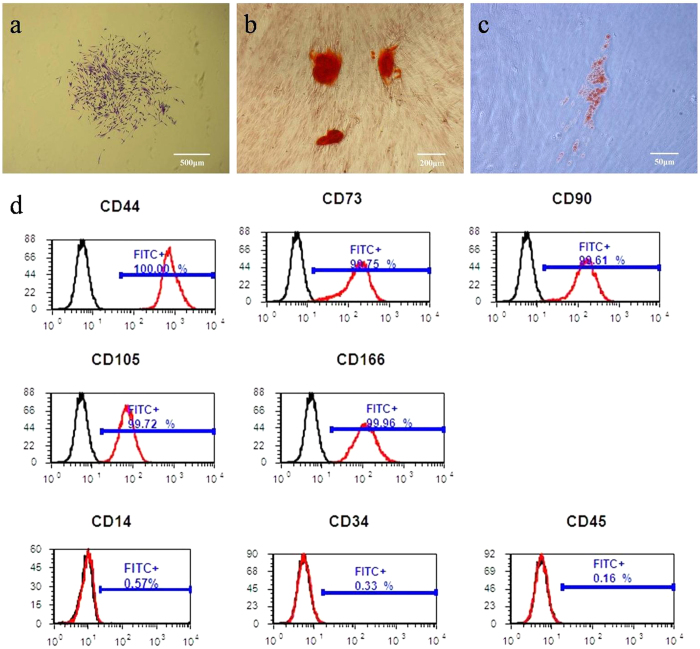
Characterization and flow cytometric analysis of human PDLSCs. (**a**) Cell clusters derived from periodontal ligament formed a single colony and were stained with 0.1% toluidine blue. (**b**) After cultured in osteogenic induction media for 4 weeks, cells formed minerals positive for Alizarin Red staining. (**c**) After cultured in adipogenic induction media for 4 weeks, cells formed lipid vacuoles positive for Oil Red O staining. (**d**) PDLSCs were positive for MSCs markers CD44, CD73, CD90, CD105 and CD166 and negative for hematopoietic markers CD14, CD34 and CD45 (red line). Isotype controls 1B5 (IgG1) and 1D4.5 (IgG2a) were black line.

Single-cell suspension from human periodontal ligament formed adherent clonogenic cell clusters in which cells exhibited typical fibroblastic morphology (Fig. 2a). This colony-forming cell population was termed as PDLSCs. The progeny of PDLSCs exhibited typical spindle-shaped fibroblast-like morphology. Moreover, after cultured in induction media for 4 weeks, PDLSCs formed minerals and lipid vacuoles (Fig. 2b,c).

### Flow cytometry analysis of human PDLSCs

Cultured human PDLSCs expressed mesenchymal stem cells (MSCs)-associated surface markers CD44, CD73, CD90, CD105, CD166, and lacked expression of hematopoietic markers CD14, CD34, CD45 (Fig. 2d).

### SEM images of human PDLSCs on scaffolds

**Figure 3:**
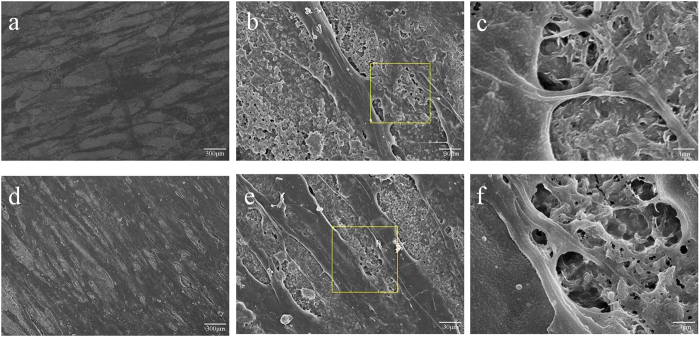
SEM images of the PDLSCs. (**a**–**c**) PDLSCs cultured on Na-Ti substrates. (**d**–**f**) PDLSCs cultured on GO-Ti substrates. (**c**,**f**) were the higher magnification of the yellow square of (**b**,**e**), respectively.

To evaluate the biocompatibility of GO-Ti substrates, the morphology of cells on Na-Ti and GO-Ti substrates was observed by SEM at day 3 (Fig. 3). PDLSCs uniformly distributed on the surface of both Na-Ti and GO-Ti substrates with fibroblast-like or spindle shape (Fig. 3a,d) and displayed many cytoplasmic lamellipodia (Fig. 3b,c,e,f).

### CLSM images of human PDLSCs on scaffolds

**Figure 4:**
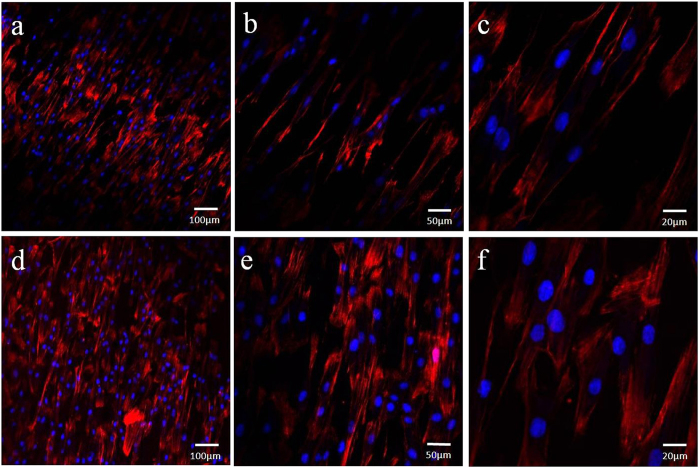
CLSM images of the PDLSCs. (**a**–**c**) PDLSCs cultured on Na-Ti substrates. (**d**–**f**) PDLSCs cultured on GO-Ti substrates. Red signals represent cells and blue signals represent cell nuclei.

The morphology and distribution of viable PDLSCs after 3 days of culture on the two substrates were observed by CLSM. F-actin filaments were stained using Alexa Fluor® 594 phalloidin, which could emit red fluorescence when excited by light at a wavelength of 594 nm. Blue signals represented cell nucleus which were excited by light at a wavelength of 405 nm (Fig. 4). From lower resolution of CLSM images, human PDLSCs on Na-Ti substrates were less dense than those on GO-Ti substrates (Fig. 4a,b,d,e).

### Cell proliferation assay

**Figure 5:**
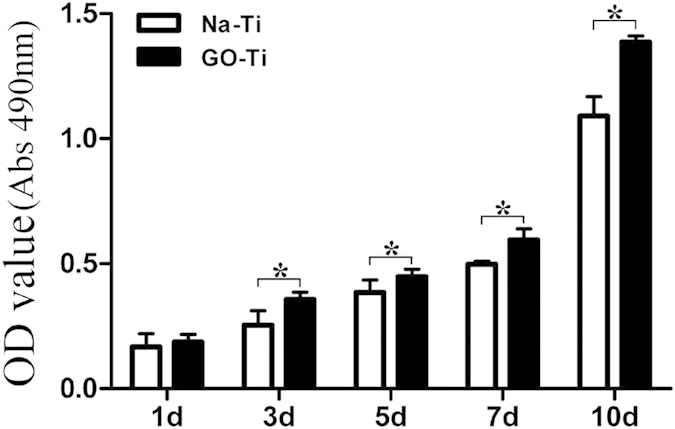
MTT assay of PDLSCs cultured on Na-Ti and GO-Ti substrates. Data represents means ± SD (n = 8, eight replicates per time-point for each experimental condition). *P < 0.05, **P < 0.01, Na-Ti *versus* GO-Ti.

An MTT assay was used to evaluate the proliferation of PDLSCs on GO-Ti and Na-Ti substrates. At day 1, there was no significant difference in cell proliferation between PDLSCs cultured on GO-Ti and Na-Ti substrates. However, 3, 5, 7 and 10 days after seeding, PDLSCs on GO-Ti proliferated at substantially higher rate than those on Na-Ti (Fig. 5).

### ALP activity

**Figure 6:**
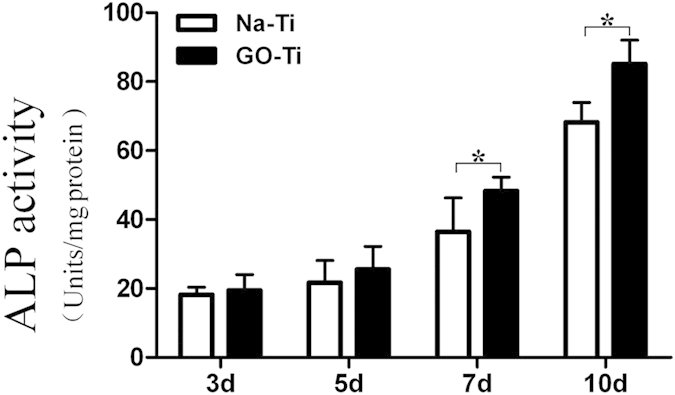
ALP activity of PDLSCs cultured on Na-Ti and GO-Ti substrates. Data represents means ± SD (n = 8, eight replicates per time-point for each experimental condition). *P < 0.05, **P <  0.01, Na-Ti *versus* GO-Ti.

ALP activity has been widely used as a marker of the early differentiation of osteoblast-like cells^32^. In our study, ALP activity of PDLSCs on two different substrates was measured for up to 10 days. At day 3 and 5, there was no significant difference in ALP activity between PDLSCs cultured on the two substrates, while at day 7 and 10, PDLSCs on GO-Ti substrates showed higher ALP activity than those on Na-Ti (Fig. 6), which suggested that the presence of GO stimulated an early stage of osteoblastic differentiation.

### Real-time PCR analysis of osteogenesis-related genes

**Figure 7:**
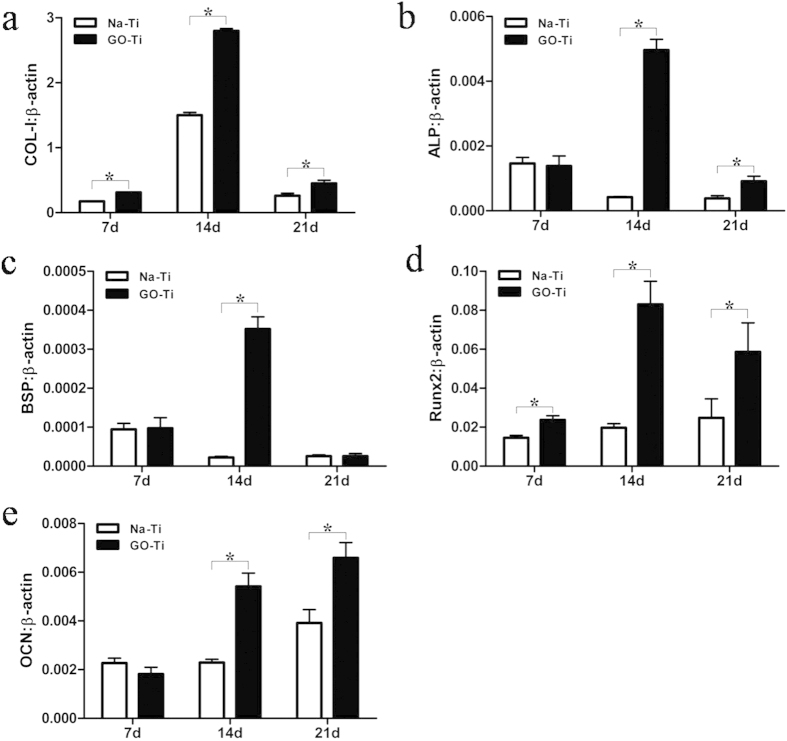
Real-time PCR analysis of COL-I, BSP, ALP, Runx2 and OCN mRNA expression in PDLSCs on Na-Ti and GO-Ti substrates. Gene expression is normalized to housekeeping gene β-actin expression. PDLSCs were cultivated for 7, 14 and 21 days on Na-Ti and GO-Ti substrates. Data represent means ± SD (n = 3). *P < 0.05, **P <  0.01, Na-Ti *versus* GO-Ti.

To further determine the effects of GO on bone formation, total RNA was isolated and RT-PCR was performed to measure the gene expression of osteogenic markers. At day 7, only the expression levels of COL-I and Runx2 were significantly elevated in GO-Ti groups compared with those in Na-Ti groups (Fig. 7d). The gene expression of COL-I, ALP, BSP and Runx2 peaked at day 14 and there was significant difference between the two groups (Fig. 7a–d). Though after 21 days culture, the gene levels of COL-I, ALP and Runx2 decreased dramatically, gene expression levels of these three genes were still significantly higher in GO-Ti groups than those in Na-Ti groups (Fig. 7a,b,d). At the same time, BSP expression level was the lowest at day 21 and there was no significant difference between GO-Ti substrates and Na-Ti substrates (Fig. 7c). As a relatively late osteogenic marker, OCN expression level peaked at day 21 and a significant difference was found between two groups at both day 14 and 21(Fig. 7e).

### Western blot analysis of bone-related proteins

**Figure 8:**
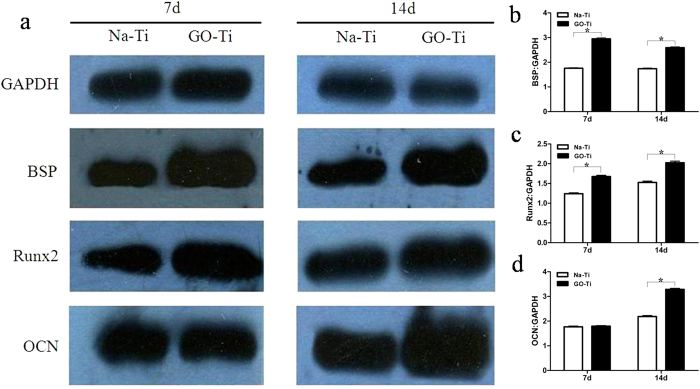
Protein expression of BSP, Runx2 and OCN in PDLSCs on Na-Ti and GO-Ti substrates. (**a**) Time-related expression of BSP, Runx2 and OCN in PDLSCs as analyzed by Western blot. (**b**) Quantitative analysis of the protein expression of BSP. (**c**) Quantitative analysis of the protein expression of Runx2. (**d**) Quantitative analysis of the protein expression of OCN. Protein expression is normalized to GAPDH expression. PDLSCs were cultivated for 7 and 14 days on Na-Ti and GO-Ti substrates. Data represent means ± SD (n = 5). *P < 0.05, **P <  0.01, Na-Ti *versus* GO-Ti.

Western blot was used to detect the protein expression of osteogenic markers. The results demonstrated that the expression of BSP and Runx2 increased at day 7 and 14, and exhibited significant increase in GO-Ti groups when compared with Na-Ti groups (Fig. 8a–c). Meanwhile, GO up-regulated the expression of OCN at day 14, and there was no significant difference between the two groups at day 7 (Fig. 8d).

## Discussion

In this study, we explored the use of GO sheets as a stem cell culture substratum to provide suitable environments for the proliferation and differentiation of human PDLSCs. To this end, we coated GO on Na-Ti using a self-assembly method by immersing the APTES-treated sodium titanate substrates with an electrostatic interaction in 0.1mg/ml GO aqueous dispersion, and the fabricated GO-Ti substrate exhibited a uniform and homogenous surface^22^. Here, GO-Ti and Na-Ti substrates have different topographical structure and provide different spatial environment and diverse cell-material interactions for PDLSCs. The surface structure of GO-Ti was characterized by SEM and different sizes of GO deposited on the titanium substrate.

To evaluate the biocompatibility of the two scaffolds, the morphology of cells on Na-Ti and GO-Ti substrates was observed under SEM and CLSM. PDLSCs maintained typical spindle shapes on the two scaffolds and displayed fine spreading with cytoplasmic lamellipodia and connections with neighbouring cells, which indicates the good biocompatibility of GO coatings. Furthermore, viable PDLSCs on the two scaffolds observed by CLSM exhibited some differences. PDLSCs on Na-Ti substrates exhibited lower density than those on GO-Ti. The results of MTT assays further confirmed that human PDLSCs on GO-Ti substrates proliferated at higher rate than those on Na-Ti substrates. The promoted effect of GO-Ti substrate on PDLSCs proliferation means that more pluripotent stem cells can grow on this scaffold, which will facilitate the regeneration of bone tissue or periodontal tissue. Both the cell morphology and the proliferation results indicated that GO coated substrates were cell-friendly and biocompatible. Biocompatibility of scaffolds is a prerequisite for generating cell-biomaterial constructs and for their successful clinical application^33^. Rodríguez-Lozano and his colleagues stated that GO plus fibroin-coated surfaces significantly improved F-actin content, cell spreading, growth rate and maintained the mesenchymal stem phenotype of PDLSCs^30^, which was in line with our results. GO-Ti substrates could promote cell attachment and cell proliferation, which might be attributed to the surface modification by GO. Dinescu *et al*. reported that the addition of GO into the polymer matrix led to a decrease of interconnected pore size and provide a massive porous structure, which is believed to be advantageous for cell attachment and proliferation allowing ingrowth of cells^34^.

Recent studies demonstrate that GO can not only affect cell shape, adhesion, migration, and proliferation, but also determining the commitment of stem cells to different lineages^19^,^20^. Our results demonstrated that PDLSCs cultured on GO-Ti substrates exhibited higher ALP activity than those on Na-Ti, which suggested that the presence of GO stimulated an early stage of osteoblastic differentiation. In Zhao’s study^24^, GO coated titanium implants could increase ALP activity and OCN secretion by MC3T3-E1 and promote MC3T3-E1 cell differentiation. In this current study, ALP activity and bone related markers were detected to evaluate the effect of GO on the osteogenic differentiation of PDLSCs. ALP is involved in dephosphorylation of collagen and an essential enzyme for calcification of osteoblasts, which is considered as an early marker of mineralized ECM^35^. Moreover, GO could also regulate the transcriptional level and gene expression of bone related markers. In our study, gene expression levels of COL-I, ALP, BSP, Runx2 and OCN were up-regulated on GO-Ti substrates. COL-I expression was selected for the biological support to which mineralized tissue nucleates and grows. BSP known as an early osteogenic marker is important in the differentiation of osteoblasts^36^,^37^, while OCN is a relatively late marker of osteoblast differentiation related to mineralization^38^. This explained why the expression of BSP reached its highest level earlier (at day 14) than OCN (at day 21) in this study. Runx2 is an osteogenic transcription factor that takes part in the process of MSCs differentiation towards osteoblasts^39^. In our results, GO promoted Runx2 expression at day 14 and day 21. Then, we subjected BSP, Runx2 and OCN to further western blot analysis at day 7 and day 14, and obtained the similar results with RT-PCR. The up-regulated proteins expression of bone-related markers further demonstrated the osteopromotive activity of GO.

The exact reason why GO-Ti substrates promote cell adhesion, proliferation and differentiation remains unknown. A combination of several factors^26^,^40^ including nanoscale structure, mechanical strength, roughness, interactions with proteins through hydrophobic and electrostatic interactions, large dosage of loading capacity may contribute to the effects of GO on the behavior of PDLSCs. GO-Ti substrates consist of hydrophobic π domains in the core region and ionized groups around GO edges. Moreover, the oxygen functional group on GO-Ti substrates introduced a negatively charged surface, which led to various interactions such as electrostatic forces, hydrogen bonding, and hydrophobic interactions between GO and cells as well as specific proteins^41^. In addition, GO could bind with insulin electrostatically^19^, suggesting that the unique properties of GO may provide suitable environments to regulate PDLSCs bioactivity of attachment, proliferation and differentiation.

Taken together, GO is believed to be a useful platform for modulating structure and function of PDLSCs. Using a self-assembly method, GO-Ti substrates were successfully fabricated and provided a suitable environment for the attachment, morphology, proliferation and differentiation of PDLSCS. Higher proliferation rate of PDLSCs on GO-Ti substrates than on Na-Ti substrates were achieved. In addition, the osteogenic differentiation of PDLSCs on GO-Ti substrate was higher compared to that on Na-Ti substrate. With the aforementioned advantages of GO, the combination of sodium titanium, GO and PDLSCs holds promise for their therapeutic use in regenerative dentistry. Further additional studies will focus on how to use GO as a biocompatible and implantable platform for the delivery of therapeutic proteins for applications related to regenerative medicine, especially for the success of dental implants. Dental implant made of GO-Ti can be combined with therapeutic protein delivery system to improve the integration of implants with bone tissues at the implantation site.

## Methods

### Preparation of sodium titanate substrates

Titanium foils (99.7% Ti TA2, 7 × 7 × 0.8 mm^3^, BaoTi Group Co. Ltd., China) were mechanically polished by water sandpaper (grades 400, 600, 800 and 1000) sequentially and then ultrasonically cleaned with acetone, ethanol and ultrapure water successively. Nano-network-structured sodium titanate thin substrates were prepared on the surface of titanium foil by the alkali-hydrothermal reaction. A typical preparation process was as follows: Titanium foils were immersed in 20 mL of 10 M NaOH aqueous solution, followed by hydrothermal treatment at 60 °C for 24 h. The as-prepared thin films were then rinsed repeatedly with ultrapure water until the pH of water dropped to 7 to obtain the “Na-Ti” substrates.

### Preparation of graphene oxide coated sodium titanium

GO was prepared from graphite powder through a modified Hummer’s method^42^. Sodium titanate films were immersed in 2% ethanol solution of 3-aminopropyltrienthoxysilane (3-APTES), and then immersed in 0.1 mg/mL GO for 24 h, washed with water for three times and dried at 37 °C. The final products, GO-coated titanium (GO-Ti) substrates were characterized by AFM (Nanowizard^®^II, JPK, Germany), SEM (S-4800, Hitachi, Japan) and Raman spectroscopy (LabRAM HR800, Jobin-Yvon, France). The samples for the characterization of Raman spectroscopy were prepared by dropping GO aqueous solution on a glass slide and then for data collection. The samples for AFM characterization were prepared by depositing GO aqueous solution on the quartz substrate and then dried in vacuum drying oven. For *in vitro* tests, Na-Ti substrates with/without GO were immersed in 70% ethanol and sterilized with UV radiation for 0.5 h on each side, and then rinsed twice with sterile ultrapure water.

### Collection and culture of human PDLSCs

All research procedures were approved by the Medical Ethics Committee of Medical School, Shandong University (approval number: 2010015; Jinan, People’s Republic of China) and written informed consent was obtained from each individual participant. All the protocols were carried out in accordance with the approved guidelines. Human PDLSCs were isolated from healthy periodontal ligaments of the premolar extracted for orthodontic reason. The teeth were stored in Dulbecco’s modified Eagle’s medium (DMEM; Hyclone, Logan, UT) supplemented with antibiotics (300 U/mL penicillin and 300 mg/mL streptomycin, Sigma-Aldrich, St Louis, MO). Human periodontal ligament tissue was scraped from the middle third of the root surface as previously described^43^. The PDL tissue was cut into small pieces and digested with 3 mg/mL collagenase I (Invitrogen, Carlsbad, CA) and 4 mg/mL dispase II (Invitrogen) for 2 h at 37 °C. The dissociated cell suspension was filtered through a 70 μm cell strainer (BD Falcon, BD Biosciences, Bedford, MA). After cell counting, single cell suspension was plated at a concentration of 60 cells/cm^2^ on nontreated 10-cm petridishes for single cell-derived colony selection, and was cultured in DMEM with 10% foetal calf serum (FCS; Hyclone, UT, USA), 2 mM L-glutamine (Sigma-Aldrich), 100 mM L-ascorbate-2-phosphate (Wako Pure Chemical Industries, Richmond, VA, USA), 1 mM sodium pyruvate (Sigma-Aldrich), 50 U/ml penicillin G and 50 mg/ml streptomycin for 10–14 days. Individual colonies were isolated with colony rings and expanded into individual vessels for further cultivation, as previously described^44^,^45^.

### Multipotent differentiation of single colony-derived PDLSCs

Osteogenic differentiation was induced as previously described^46^. PDLSCs were plated at 8 × 10^3^ cells/well in 96-well plates and cultured in DMEM supplemented with 5% FCS, 100 mM L-ascorbate-2-phosphate, 1 mM sodium pyruvate, 50 μg/ml streptomycin, 50 U/ml penicillin G, 2 mM L-glutamine, 0.1 μM dexamethasone and 1.8 mM inorganic phosphate for 4 weeks with medium changed twice weekly. At 28 days, wells were washed three times with PBS and cells were fixed with 10% neutral buffered formalin for 1 h at room temperature. Mineral deposit formation was identified by staining with 2% Alizarin Red S (Sigma-Aldrich). Adipogenic differentiation was induced as previously described^46^. PDLSCs were plated in 96-well plates and cultured in DMEM supplemented with 10% FCS, 100mM L-ascorbate-2-phosphate, 1 mM sodium pyruvate, 50 μg/ml streptomycin, 50 U/ml penicillin G, 2 mM L-glutamine, 0.1 μM dexamethasone and 60 μM indomethacin (Sigma-Aldrich) for 4 weeks with medium changes twice a week. At 28 days, cells were fixed and formation of lipid-laden fat cells was determined by staining with 0.5% (w/v) Oil Red O (MP Biomedicals, Solon, OH, USA).

### Flow cytometric analysis

PDLSCs were detached with 0.05% trypsin/EDTA and resuspended in blocking buffer containing Hanks’ Balanced Salt Solution (Sigma-Aldrich) supplemented with 5% FCS, 1% BSA and 5% normal human serum for one hour. Approximately 1 × 10^5^ cells were incubated with specific FITC-conjugated mouse monoclonal antibodies(10 μg/ml) for human CD44, CD73, CD90, CD105, CD146 (Becton Dickinson Biosciences, San Jose, CA), CD14, CD34, CD45 (Beckman Coulter, Fullerton, CA, USA) or 10 μg/ml isotype-matched control 1B5 (IgG_1_) and 1D4.5 (IgG_2a_). After incubation on ice for 1 h, cells were incubated with goat anti-mouse IgG or IgM for 45 min on ice. After washing, flow cytometry data were analyzed using an Epics-XL/MCL flow cytometer (Beckman Coulter, Hialeah, FL, USA).

### SEM observation

Na-Ti substrates with/without GO were placed in 48-well plates and seeded with 500 μL cell suspension containing 1 × 10^4^ human PDLSCs. The culture medium changed every other day. After 3 days, the cell-seeded samples were removed from the media and gently rinsed with phosphate-buffered solution (PBS; Hyclone). The cells cultured on the substrates were then fixed with 2.5% glutaraldehyde solution for 1 h at 4 °C. After removing the fixative, the foils were subsequently rinsed gently with PBS and ultrapure water, and were then dehydrated for 10 min with ethanol at a series of concentrations (30, 50, 70, 80, 90, and 100%). Afterwards, the samples were dried for 1 day and coated with platinum for 40 seconds prior to SEM examination at an accelerating voltage of 5 kV.

### CLSM observation

Three days after initial seeding, the cell-coated substrates were fixed with 3.7% formaldehyde solution for 10 min and then extracted with 0.1% Triton-X100 (Sigma-Aldrich) for 5 min, blocked with 1% bovine serum albumin (BSA; Sigma-Aldrich) for 30 min, stained with phalloidin conjugated to Alexa Fluor 594® (Invitrogen), and examined at excitation wave lengths of 594 nm and 405 nm with a confocal laser scanning microscope (CLSM; LSM780, Zeiss, Germany).

### MTT assays

Proliferation of PDLSCs cultured on Na-Ti and GO-Ti substrates was determined by 3-(4, 5-dimethylthiazol-2-y1)-2, 5-diphenyltetrazolium bromide (MTT; Sigma-Aldrich) assay. Cells were cultured on the two substrates and employed for quantitative evaluation of cell proliferation after incubation for 1, 3, 5, 7 and 10 days. For quantitation, 20 μL MTT (5 mg/mL) was added into each well and incubated at 37 °C for 4 h. The medium was removed and 200 μL dimethylsulfoxide (DMSO; Sigma-Aldrich) was added, and then 150 μL solution was extracted to 96-well after shaking for 10 min. The absorbance value was measured in a multi-well Spectrophotometer (Bio-Rad, Hercules, CA) at 490 nm.

### ALP activity

PDLSCs were seeded on Na-Ti and GO-Ti substrates at the concentration of 5 × l0^4^ cells/mL. After 3, 5, 7 and 10 days of induction, cells were washed with 0.01 M PBS and scraped into 100 μL 0.2% Triton-X100. Then cells were sonicated and cell lysates were centrifuged at 14,000 g for 10 min at 4 °C. Following the manufacturer’s instructions, ALP activity was measured using an ALP activity assay kit (Beyotime Biotech, Jiangsu, China). ALP activity was calculated using p-nitrophenol as a standard, according to the instructions given in the kit and was expressed as ALP Units’mg^−1^of protein. Absorbance measurements at 405 nm were normalized to total protein content measured using BCA protein assay (Beyotime Biotech).

### RNA isolation & real-time PCR analysis

After 7, 14 and 21 days of culture, total RNA of PDLSCs were isolated using TRIZOL (Invitrogen). 3 μg of RNA was reverse transcribed to the complementary DNA using Reverse Tanscriptase (TaKaRa Bio, Mountain View, CA), and 20 ng of complementary DNA (total volume of 20 μL) was used for DNA amplification. The amount of mRNA was normalized by β-actin level. The sequences of the primers for amplification of COL-I, ALP, BSP, Runx2 and OCN were in Table 1. Real-time PCR was performed with RT2 SYBR Green/ROX qPCR Master Mix (TaKaRa Bio). Cycling parameters used were: 30 seconds at 95 °C, 45 cycles of 5 seconds at 95 °C, 35 seconds at 60 °C, 15 seconds at 95 °C, 1 min at 60 °C and final extension held 30 seconds at 40 °C.

### Western blot analysis

After culturing for 7 and 14 days, cells were washed with PBS and lysed using a RIPA lysis buffer (Sigma-Aldrich). 30 μg of lysate protein samples were loaded to SDS-PAGE gels and transferred to nitrocellulose membranes. Membranes were blotted with primary antibodies overnight at 4 °C and subsequently with secondary antibodies (Beyotime Biotech) for 1 h at room temperature. The proteins were visualized using enhanced chemiluminescence reagents from Pierce Biotechnology (Rockford, IL, USA). Image J (softonic^®^) software was used to qualify the protein expression. The primary antibodies used were as follows: anti-BSP (AB52128, Abcam, Cambridge, MA), anti- Runx2 (AB76956, Abcam), and anti-OCN (AB13420, Abcam).

### Statistical analysis

The data are expressed as the means ± SD. All experiments were repeated at least three times and Student’s *t-test* was performed to test the significance of results. All data were considered statistically significant when *P* < 0.05.

## Additional Information

**How to cite this article**: Zhou, Q. *et al*. Bioactivity of periodontal ligament stem cells on sodium titanate coated with graphene oxide. *Sci. Rep*. **6**, 19343; doi: 10.1038/srep19343 (2016).

## Figures and Tables

**Table 1:** Primer sequences.

Gene	GenBank no	Product size (bp)	Forward primer 5′-3′	Reverse primer 5′-3′
β-actin	NM_001101.3	173	AGCCTCGCCTTTGCCGA	CTGGTGCCTGGGGCG
COL-I	XM_005257059	26	TTTGTGGACCTCCGGCTC	AAGCAGAGCACTCGCCCT
ALP	NM_001632.4	170	AGTGCAGCTCATACTCCATGC	GCGGTTCCAGAAGTCCGGGTT
BSP	NM_004967.3	199	CTGGCACAGGGTATACAGGGTTAG	GCCTCTGTGCTGTTGGTACTGGT
Runx2	NM_011514964	135	TCCACACCATTAGGGACCATC	TGCTAATGCTTCGTGTTTCCA
OCN	NM_001199662	223	AGGGCAGCGAGGTAGTGAAG	CTCCTGAAAGCCGATGTGGT

Abbreviations: COL-I, collagen type I; ALP, alkaline phosphatase; BSP, bone sialoprotein; Runx2, runt-related transcription factor 2; OCN, osteocalcin.
